# Therapeutic efficacy of artemether–lumefantrine for the treatment of uncomplicated *Plasmodium falciparum* malaria from three highly malarious states in India

**DOI:** 10.1186/s12936-016-1555-4

**Published:** 2016-10-13

**Authors:** Praveen K. Bharti, Man M. Shukla, Pascal Ringwald, Sri Krishna, Pushpendra P. Singh, Ajay Yadav, Sweta Mishra, Usha Gahlot, Jai P. Malaiya, Amit Kumar, Shambhu Prasad, Pradeep Baghel, Mohan Singh, Jaiprakash Vadadi, Mrigendra P. Singh, Maria Dorina G. Bustos, Leonard I. Ortega, Eva-Maria Christophel, Sher S. Kashyotia, Gagan S. Sonal, Neeru Singh

**Affiliations:** 1 National Institute for Research in Tribal Health (NIRTH), Jabalpur, Madhya Pradesh 482003 India; 2 Global Malaria Programme World Health Organization, Geneva, Switzerland; 3 Community Health Centre Ranapur, District Jhabua, Madhya Pradesh India; 4 Community Health Centre Rajendragram, District Anuppur, Madhya Pradesh India; 5 Community Health Centre Jaldega, District Simdega, Jharkhand India; 6 Community Health Centre Bano, District Simdega, Jharkhand India; 7 Community Health Centre Kilepal, District Bastar, Chhattisgarh India; 8 Satpura Bhawan, Bhopal, Madhya Pradesh India; 9 Indravati Bhawan, Raipur, Chhattisgarh India; 10 National Institute of Malaria Research (NIMR) Field Station, Jabalpur, MP India; 11 World Health Organization, Country Office for Thailand, Bangkok, Thailand; 12 World Health Organization, Regional Office for South-East Asia, New Delhi, India; 13 National Vector Borne Disease Control Programme (NVBDCP), New Delhi, India

**Keywords:** Therapeutic efficacy, Artemether–lumefantrine, *Plasmodium falciparum*, Malaria, India

## Abstract

**Background:**

Anti-malarial drug resistance continues to be a leading threat to malaria control efforts and calls for continued monitoring of waning efficacy of artemisinin-based combination therapy (ACT). Artesunate + sulfadoxine/pyrimethamine (AS + SP) is used for the treatment of uncomplicated *Plasmodium falciparum* malaria in India. However, resistance against AS + SP is emerged in northeastern states. Therefore, artemether–lumefantrine (AL) is the recommended first line treatment for falciparum malaria in north eastern states. This study investigates the therapeutic efficacy and safety of AL for the treatment of uncomplicated falciparum malaria in three malaria-endemic states in India. The data generated through this study will benefit the immediate implementation of second-line ACT as and when required.

**Methods:**

This was a one-arm prospective evaluation of clinical and parasitological responses for uncomplicated falciparum malaria using WHO protocol. Patients diagnosed with uncomplicated mono *P. falciparum* infection were administered six-dose regimen of AL over 3 days and subsequent follow-up was carried out up to 28 days. Molecular markers *msp*-*1* and *msp*-2 were used to differentiate recrudescence and re-infection and K13 propeller gene was amplified and sequenced covering the codon 450–680.

**Results:**

A total of 402 eligible patients were enrolled in the study from all four sites. Overall, adequate clinical and parasitological response (ACPR) was 98 % without PCR correction and 99 % with PCR correction. At three study sites, ACPR rates were 100 %, while at Bastar, cure rate was 92.5 % on day 28. No early treatment failure was found. The PCR-corrected endpoint finding confirmed that one late clinical failure (LCF) and two late parasitological failures (LPF) were recrudescences. The PCR corrected cure rate was 96.5 %. The mean fever clearance time was 27.2 h ± 8.2 (24–48 h) and the mean parasite clearance time was 30.1 h ± 11.0 (24–72 h). Additionally, no adverse event was recorded. Analysis of total 186 samples revealed a mutation in the *k13* gene along with non-synonymous mutation at codon M579T in three (1.6 %) samples.

**Conclusion:**

AL is an efficacious drug for the treatment of uncomplicated falciparum malaria. However, regular monitoring of AL is required in view of malaria elimination initiatives, which will be largely dependent on therapeutic interventions, regular surveillance and targeted vector control.

**Electronic supplementary material:**

The online version of this article (doi:10.1186/s12936-016-1555-4) contains supplementary material, which is available to authorized users.

## Background

Malaria is a major public health problem and a leading cause of mortality worldwide. The World Health Organization (WHO) estimated that 214 million cases of malaria occurred globally and approximately 438,000 deaths were recorded in 2015 [1]. Globally, India is one of the 15 countries which accounted for 80 % of malaria cases and 78 % of malaria deaths in 2015 [1]. Approximately 91 % of India’s population lives in a malarious area, with 14 % of the population residing in a high-transmission area [1]. Importantly, malaria is a major health problem in rural/tribal areas of central, eastern and north eastern states of India [2]. *Plasmodium falciparum* infection causes the most serious form of malaria and its proportion varies from 50 to 90 % in different states of the country. This study was undertaken in three out of five highly malarious states, i.e., Chhattisgarh, Madhya Pradesh and Jharkhand.

The emergence of *P. falciparum* resistance to chloroquine (CQ) and sulfadoxine–pyrimethamine (SP) has been a major obstacle to malaria control and is responsible for the spread across most parts of the country [3–5]. WHO has recommended artemisinin-based combination therapy (ACT) for the management of uncomplicated malaria cases. ACT reduces both malaria-related morbidity and mortality and the transmission of *P. falciparum* by acting on gametocytes and reducing the chances of drug resistant development [6]. The National Drug Policy was revised in 2010 and since then AS + SP has become the first line for treatment of uncomplicated falciparum malaria in the country [7]. Recent studies revealed reduced AS + SP susceptibility, particularly in the north eastern states of India [8]; subsequently AS + SP treatment has been replaced with artemether–lumefantrine (AL) therapy in these areas as per revised National Drug Policy in the year 2013 [9].

AL is a co-formulation of artemether and lumefantrine (an aryl alcohol related to quinine, mefloquine and halofantrine) and is a commercially available fixed dose combination. In this combination, artemether, being a fast-acting drug, quickly reduces the parasite biomass [10] and resolves the clinical symptoms, while long-acting lumefantrine prevents recrudescence. This dual effect eventually reduces the selective pressure on the parasite to develop resistance. AL has been available commercially in India since 2006 and is being used by the private sector [11]. Currently 40 countries in Africa and six countries in South America are using AL as their first- or second-line treatment [1].

Recently polymorphisms in the propeller region of *P. falciparum* Kelch protein (*k13* gene) have been found to be associated with artemisinin resistance [12]. After this established association, WHO included *k13* mutations in its new working definition for partial artemisinin resistance [13]. In order to ensure effective malaria case management, it may be imperative to preserve the user life of ACT. WHO recommends a regular efficacy monitoring by all malaria-endemic countries that have deployed ACT. One case of AL resistance was recorded in Odisha state, India [11].

The present study was conducted to evaluate the AL efficacy with six-dose regimen at four different sites in three states of India using the WHO therapeutic efficacy protocols. Based on these data, a second-line ACT will be available for implementation in the national programme as and when required.

## Methods

### Study design and sites

**Fig. 1:**
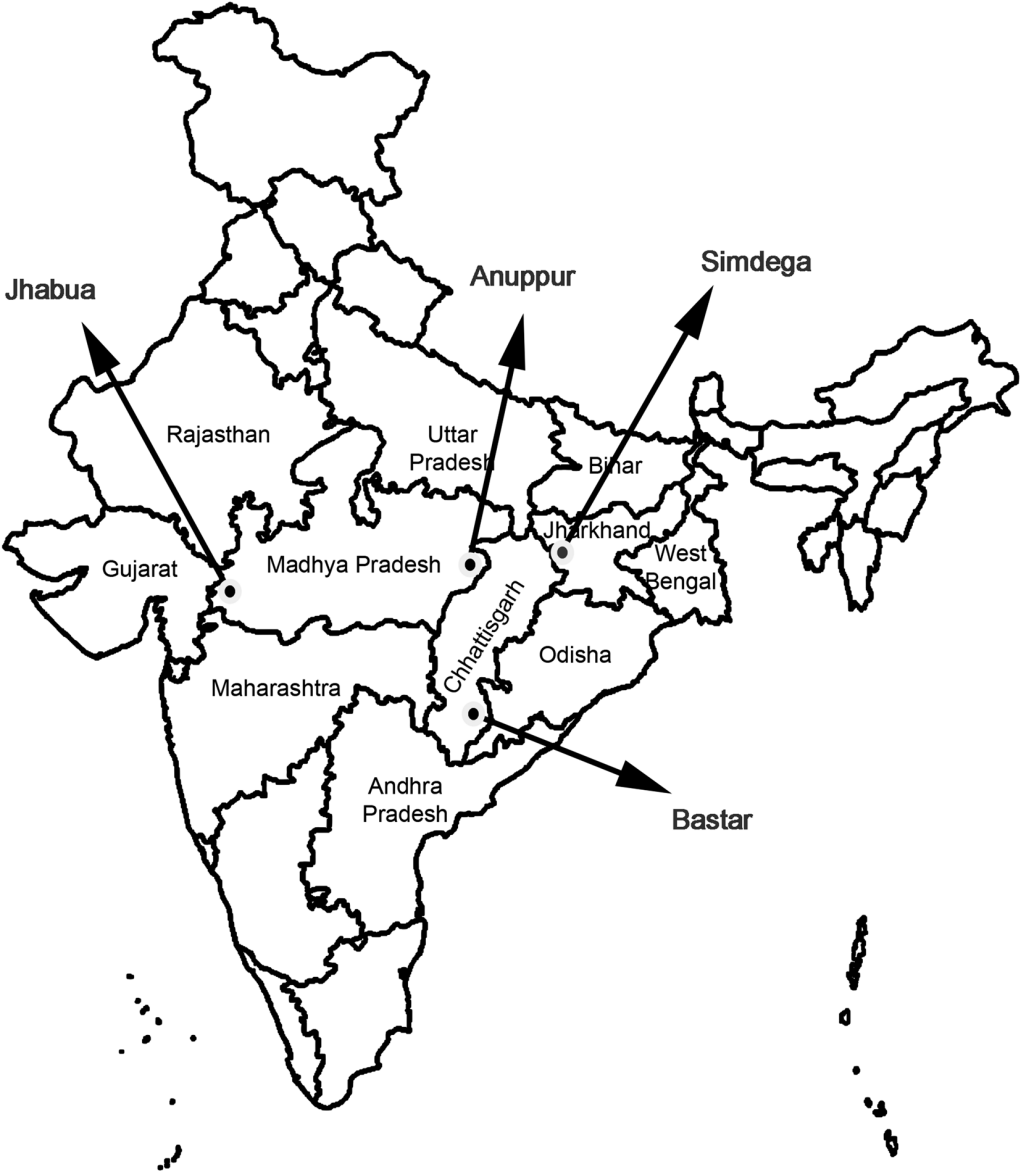
Map showing the study sites. District Jhabua and district Anuppur in Madhya Pradesh (MP); district Simdega in Jharkhand (JH); district Bastar in Chhattisgarh (CG) states

This was a one-arm prospective study conducted from October 2014 to August 2015. The study assessed clinical and parasitological responses after AL administration to eligible patients. The primary endpoint was the 28-day cure rate. This study was conducted at four Community Health Centres (CHCs) located in Anuppur and Jhabua districts of Madhya Pradesh, Simdega district of Jharkhand, and Bastar district of Chhattisgarh, which borders three states, i.e., Maharashtra, Odisha and Andhra Pradesh (Fig. 1). These sites were selected based on their malaria epidemiological and geographic profile. *Plasmodium falciparum* and *Plasmodium vivax* are the two dominant parasitic species with relative frequencies of about 70 and 30 %, respectively, (although this value may vary according to location and season). The selected CHCs had sufficient facilities in terms of human resources and were well equipped with all required facilities for management of uncomplicated malaria. All CHCs were situated near a secondary- or tertiary-level district hospital for referrals of severe malaria case management, if required.

### Study population

Consenting patients with uncomplicated falciparum malaria seeking care at the selected CHCs, who were aged 6 months and above (>5 kg body weight), were enrolled in the study. Women in the age group 12–18 years were excluded as a request for a pregnancy test or initiation of contraceptives is not acceptable within the local population.

### Inclusion criteria

Symptomatic patients aged 6 months and above (>5 kg body weight) with uncomplicated malaria due to mono-infection of *P. falciparum* (detected by microscopy at parasitaemia of 1000 to 100,000/μL asexual forms, axillary temperature ≥37.5 °C) and willing to comply with the study protocol for the duration of the study were included [14].

### Exclusion criteria

Patients with general danger signs or signs of severe falciparum malaria, who were unable to drink, had severe vomiting, reported a history of convulsion 7 days prior to patient contact, presence of lethargy or decreased consciousness, inability to sit or stand, were all excluded. Patients who failed to complete treatment due to persistent vomiting or failed to attend scheduled visits during the first 3 days or withdrew their consent were also excluded [14].

### Pregnancy test

Female patients of child-bearing age, defined as those who menstruate and are sexually active, were asked to take a urine pregnancy test before enrolment in the study, because the AL is contra-indicated during the first trimester of pregnancy. These patients were also asked to take a urine pregnancy test on the 28th day follow up for analysis purpose.

### Procedures

After obtaining the informed written consent from patients or guardian, complete patient medical history (symptoms, current medications and previous use of anti-malarial drugs) was noted. A complete physical examination was performed and case record form was filled in for each patient. All enrolled patients were treated with AL on site, as directly observed treatment and monitored for 28 days [14]. Follow-ups were carried out on days 1, 2, 3, 7, 14, 21, and 28. On each follow-up, clinical signs and symptoms of malaria were recorded, blood smears were performed for detecting the malaria parasites, and filter-paper spot samples were taken for genotyping.

### Anti-malarial treatment

AL was obtained from the WHO Headquarter batch no. DYI573943, expiry date 09/2015 and administered by a Medical Officer based on the patient’s weight [6]. The day a patient was enrolled and received the first dose of AL was designated as ‘day 0’. A second dose was administered after 8 h and subsequent two doses daily was administered on the second and third day. Antimalarial was given with some food. Enrolled patients were observed for a minimum of 30 min after treatment to ensure that they did not vomit the drugs out. Patients with persistent vomiting were excluded from the study and referred to the district hospital for appropriate management. Patients were advised not to take any herbal remedies during the study to avoid effects that would confound interpretation and lead to false results.

### Microscopic blood examination

Parasite counts were done on Giemsa-stained thick films and the numbers of parasites per 200 white blood cells (WBCs) were counted by light microscopy. Parasite density, expressed as the number of asexual parasites per μL of blood, was calculated by dividing the number of asexual parasites by the number of WBCs counted, and then multiplying it by an assumed WBC density (typically 6000 per μL). When the number of asexual parasites was fewer than 100 per 200 WBCs in follow-up smears, counting was done against at least 500 WBCs. A blood-slide sample was considered negative when examination of 1000 WBCs or 100 fields containing at least ten WBCs per field revealed no asexual parasites. The presence of gametocytes on the day the patient was enrolled or on the day of follow-up was also recorded.

### Quality assurance of microscopy

Blood smears of enrolled patients, including follow-up smears, were examined by two independent microscopists [one at each study site and another at the National Institute for Research in Tribal Health (NIRTH) Laboratory, Jabalpur]. If any discordance was found, a third reading was performed by another senior microscopist. Quantification of parasitaemia was also performed by two independent microscopists with a third reading performed by a senior microscopist if the difference between the first two readings varied by more than 25 %. Each reader was blinded to the result of other reader.

### Polymerase chain reaction (PCR)

The PCR method was used to distinguish recrudescence from new infection in the case of treatment failure. Nested PCR was conducted to compare the genetic polymorphism of *P. falciparum* genes merozoite surface proteins (*msp1* and *msp2*) [3]. Recrudescence was defined as at least one identical allele for each of the two markers in the pre-treatment and post-treatment samples. New infections were diagnosed when all alleles for at least one of the markers differed between the two samples.

### Follow-up and loss to follow-up

Parents or guardians of children were instructed to return to the health centre at any time if they had any general danger signs as described under exclusion criteria. The study team made home visits as follow-ups for study participants that were late for their scheduled visits. Patients who failed to return on days 1 and 2 and missed one dose of the treatment or enrolled patients who could not attend scheduled visits were considered lost to follow-up (LFU) and excluded from the final analysis.

### Classification of responses to treatment

On the basis of parasitological and clinical outcome of treatment with AL, patients were classified according to the WHO definition of therapeutic responses: early treatment failure (ETF), late clinical failure (LCF), late parasitological failure (LPF) and adequate clinical and parasitological response (ACPR) [14].

### *k13* propeller gene amplification and sequencing

The *k13* propeller gene was amplified by the nested PCR method described earlier by Ariey et al. [12] with some modifications. The cycling conditions for the first round were as follows: an initial denaturation step at 94 °C for 5 min; 35 cycles of denaturation at 94 °C for 30 s, annealing at 60 °C for 90 s and extension at 72 °C for 90 s; followed by a final extension at 72 °C for 10 min. The cycling conditions for the second round were same as first round for 30 cycles. All amplification reactions were carried out in a final volume of 25 μL. An aliquot of the PCR products was analysed by electrophoresis on a 1.5 % agarose gel stained with ethidium bromide to confirm amplification. The capillary sequencing of 849 bp (1279–2127) PCR products was performed by means of Sanger sequencer standard methods, using the Applied Biosystems 3130XL system. The sequences were analysed using software Bioedit Sequence Alignment Editor v. 7.0.5.2 and aligned with sequences of PF3D7 1343700 Kelch protein propeller domain using the online sequence alignment tool ClustalW. The nucleotide sequence of *k13* gene of *P. falciparum* isolate from the study site has been submitted to GenBank under Accession number KX121048, KX121049.

### Data analysis

Data from both clinical and parasitological assessments for each participant were entered into the WHO standardized Microsoft Excel data collection sheet. This form was used for both data management and analysis. All data were independently entered double blind. Data were analysed by estimation of difference in proportion according to a 95 % confidence interval. Groups were compared using Chi Square test or Fisher Exact test for categorical variables and Student’s t test for continuous variables where ever applicable. Otherwise, non-parametric tests (Mann–Whitney, Kruskal–Wallis) were used.

## Results

### Characteristics of the study population

A total of 402 eligible patients (61 % males and 39 % females) were enrolled in the study from all four sites and the baseline demographics are presented in Table 1. The highest number of patients (228, 57 %) was from the age group of 5–15 years, while 128 patients (32 %) were older children and adults (age >15 years), with the age group under 5 years representing the lowest number of patients (46.11 %). The mean age of the study population was highest from Anuppur at 21 years ± 18.5 (0.8–60 years) and lowest from Jhabua at 12 years ± 11.3 (1–58 years). The mean body weight was almost the same from all sites (mean body weight range: 27–30 kg). The geometric mean of parasite density was relatively higher at 7868.93/μL (95 % CI 6430.46–9629.19) in Bastar and lowest at 2518.11/μL in Anuppur (95 % CI 2015.28–3146.39).

**Table 1:** Demographic and baseline characteristics of the enrolled patients

	Jhabua, MP (n = 98)	Anuppur, MP (n = 89)	Bastar, CG (n = 123)	Simdega, JH (n = 92)	Total
Age (years)					
Mean	12.12	21.03	14.72	17.02	16.01
sd	11.34	18.55	11.52	15.81	14.61
Min	1	0.83	2	2	0.83
Max	58	60	49	60	60
Age group (years) (%)					
Under 5	16 (16.23)	10 (11.24)	11 (8.94)	9 (9.78)	46 (11.44)
5–15	59 (60.20)	42 (47.19)	72 (58.54)	55 (59.78)	228 (56.72)
Adult	23 (23.47)	37 (41.57)	40 (32.52)	28 (30.43)	128 (31.84)
Sex					
Male (%)	67 (68.37)	47 (52.81)	80 (65.04)	53 (57.61)	247 (61.44)
Female (%)	31 (31.63)	42 (47.19)	43 (34.96)	39 (42.39)	155 (38.56)
Weight (kg)					
Mean	26.84	30.2	30.7	28.06	29.04
sd	13.66	15.82	15.88	14.73	15.10
Min	8	5	9	9	5
Max	67	64	64	65	67
Height (cm)					
Mean	127.98	133.98	132.36	130	131.11
sd	26.33	26.16	27.59	25.51	26.47
Min	37	65	60	65	37
Max	175	173	170	168.9	175
Parasite density/µL (at enrollment)					
Geometric mean	6868.41	2518.11	7868.93	3031.62	4750.77
95 % CI	(5755.9–8195.9)	(2015.3–3146.4)	(6430.5–9629.2)	(2086.4–4405.1)	(4163.18–5421.30)
<2000/µL n (%)	9 (9.2)	45 (50.6)	15 (12.2)	22 (23.9)	91 (22.64)
>2000/µL n (%)	30 (30.6)	27 (30.3)	29 (23.6)	33 (35.9)	119 (29.60)
>5000/µL n (%)	28 (28.6)	6 (6.7)	27 (21.9)	16 (17.4)	77 (19.15)
>10,000/µL n (%)	31 (31.6)	11 (12.4)	52 (42.3)	21 (22.8)	115 (28.61)
Gametocyte carriage					
n (%)	7 (7.1)	10 (11.2)	2 (1.6)	8 (8.7)	27 (6.72)

### Therapeutic efficacy

Among a total of 402 patients (see Additional file 1), 24 patients were lost to follow-up because of high population movement in connection with wage earning and could not be traced; 42 patients withdrew from the study mainly as they were not taken complete treatment. Out of the remaining 336 subjects, three (0.89 %; 95 % CI 0.18–2.50) patients were recorded as LCF (one on day 21 and two on day 28), four (1.19 %; 95 % CI 0.32–3.02) patients as LPF (all on day 28) and the remaining 329 (97.92 %; 95 % CI 95.75–99.16) patients were classified as ACPR (Table 2). There were no ETF and the clinical cure rate was 98 %. All four cases of LPF and two cases of LCF were in the age-group of 5–15 years whereas the sole case LCF was an adult patient. All these LCF and LPF cases were reported from Bastar, Chhattisgarh. No adverse event was recorded. Participants with treatment failure were given an alternative treatment of AS + SP as per national guideline. The PCR corrected endpoint findings confirmed one LCF (7 years) and two LPF (both 6 years) patients having *P. falciparum* recrudescence, while the remaining two LCF and one LPF patients were confirmed as having *P. falciparum* re-infection with one LPF shown as PCR negative. The PCR-corrected cure rate by Kaplan–Meier analysis at Bastar was 96.5 %. Merozoite surface protein 1 (*Pfmsp 1*) and merozoite surface protein 2 (*Pfmsp 2*) genotyping was performed for all the treatment failure samples on day 0 and day of failure. Out of the seven cases, six were found positive for *P. falciparum* on both days (day 0 and day of failure). Two cases were recrudescence (same allele for *msp1* and *msp2)* whereas three patients having re-infection at the time of treatment failure. However, one patient had mixed *msp2* allele at the time of treatment failure.

**Table 2:** Treatment outcomes by day 28, fever and parasite clearance times with AL by study site

Variables	Study sites				Total
	Jhabua (MP)	Anuppur (MP)	Bastar (CG)	Simdega (JH)	
PCR unadjusted					
Early treatment failure	0	0	0	0	0
Late clinical failure	0	0	3	0	3
Late parasitological failure	0	0	4	0	4
Adequate clinical and parasitological response (%)	80 (100 %)	75 (100)	86 (92.5)	88 (100)	329 (97.9)
PCR adjusted					
Early treatment failure	0	0	0	0	0
Late clinical failure	0	0	1	0	1
Late parasitological failure	0	0	2	0	2
Adequate clinical and parasitological response (%)	80	75	86 (95.6)	88 (100)	329 (99.1)
Pf recrudescence	0	0	2	0	2
Pf re-infection	0	0	3	0	3
Mixed with Pf recrudescence	0	0	1	0	1
PCR negative (unknown)	0	0	1	0	1
Fever clearance time (hours)					
Mean	27.4	24.0	26.1	28.8	27.7
sd	8.4	0.0	6.8	9.7	8.1
Min	24	24	24	24	24
Max	48	24	48	48	48
Parasite clearance time (hours)					
Mean	25.5	31.3	32.9	30.0	30.1
sd	5.8	12.7	12.1	10.5	10.9
Min	24	24	24	24	24
Max	48	48	72	48	72

### Fever, parasite and gametocyte clearance

At the time of enrolment, 63 % of the patients had fever. The mean fever clearance time was 27.2 h ± 8.2 (24–48 h) (Table 2). For all study subjects, fever subsided within 2 days. The mean parasite clearance time was 30.1 h ± 11.0 (24–72 h). By day 3, all study subjects were cleared for asexual parasites. Gametocytes were detected in 6.7 % (n = 27) of the patients on day 0 and all these patients cleared gametocytes by day 14.

### *Plasmodium falciparum k13*-propeller mutation

A total of 243 samples were used to analyse the mutation in *k13* gene. Out of 243 samples, sequencing of the propeller region of *k13* gene was done in 186 isolates (Table 3). After alignment with PF3D7 1343700, non-synonymous mutations (NS) were found in three (1.6 %) samples at codon M579T while the rest of the 183 sequences were perfectly aligned (codons 450–680) with wild type. All three samples were from the state of Madhya Pradesh. Treatment responses were good in each of the three patients harbouring NS *k13* mutations.

**Table 3:** Distribution of samples and non-synonymous mutations

State	Study site	Samples (n)	K13 sequencing n (%)	Non-synonymous mutation n (%)
Chhattisgarh	Bastar	93	79 (85)	0 (0.0)
Madhya Pradesh	Anuppur	50	32 (64.0)	1 (3.1)
	Jhabua	50	38 (76.0)	2 (5.2)
Jharkhand	Jaldega	50	37 (74.0)	0 (0.0)
Total		243	186 (74.4)	3 (1.6)

## Discussion

This study was carried out in three states (Chhattisgarh, Madhya Pradesh and Jharkhand), which are classified as the second, third and fourth highest malarious states, due to their high contribution in total malaria cases (11.0, 8.6 and 8.01 %, respectively) within the country [2]. The present ACT (AS + SP) recommended by the national programme is highly effective in most parts of the country, but available as a blister pack and not a fixed dose formulation. AL is the fixed dose combination that has been approved for marketing. This study has demonstrated that overall AL is highly effective for the treatment of uncomplicated falciparum malaria in all ages and in areas prone to high malaria transmission. Clinical improvement with AL was swift with rapid fever clearance. Gametocytic clearance was observed in all 27 participants by the fourteenth day with all of them becoming gametocyte-free by day 28, similar to the previous report [15]. The aggregated mean cure rate was 98 % without PCR correction and 99 % with PCR correction. This finding is consistent with the therapeutic efficacy values reported worldwide, including India [11, 15–17]. In Ethiopia, AL remains highly effective in the treatment of uncomplicated falciparum malaria and reduced gametocyte carriage even 8 years after its introduction [18].

In this study, three cases of treatment failure on day 21 and day 28 were young children. It is not known whether this is due to actual resistance of parasites to drugs or due to inadequate blood levels caused by poor drug absorption or altered pharmacokinetics. The study has limitation as the characterization the recrudescence vs. re-infection is based on only two population marker gene (*msp1* and *msp2*) and not all the three marker genes (*msp, msp2* and *Pfglurp).* All three cases of treatment failure were from Bastar, a highly malarious district bordering Odisha state, which contributes the highest number of malaria cases and malarial deaths in the country [2]. It is worthwhile to mention that the first case of AL resistance was also found in Odisha [11]. Studies carried out in a tertiary hospital in Bastar revealed that *P. falciparum* causes cerebral and severe malaria with a case fatality rate of 32 and 9 %, respectively, in PCR-confirmed mono *P. falciparum* among hospitalized patients [19]. Since falciparum malaria was significantly more prevalent in children than adults, the recrudescence of parasites on days 21 and 28 in this district led to investigate possible drug misuse, since AL was distributed without prescription by local chemists. Free availability of drugs and its misuse raises concerns of developing drug resistance in this area, where malnourished tribal people constitute more than 70 % of the population and where the *P. falciparum* proportion is more than 85 % [19]. As malnutrition and poor uptake of fat is common in poverty-stricken tribal populations, care should be taken to prescribe drugs with fatty food to ensure good absorption.

In the present study, NS gene among three isolates was detected in the highly conserved K13 propeller gene of *P. falciparum* parasites. As definite phenotype of ACT resistance is very rare, the association of polymorphism in marker genes is very difficult to correlate with the efficacy outcome. Ariey et al. have reported that polymorphisms C580Y, Y493H and R539T in the propeller region of *k13* gene are strongly associated with artemisinin resistance [12]. Following this discovery, several researchers reported a number of single nucleotide polymorphism (SNPs) in this gene, particularly at the propeller region, from different parts of the world [20–26]. Three samples from this study showed mutation at M579T codon, which is not directly linked with resistance but is located adjacent to the C580Y mutation responsible for delayed parasite clearance [12]. No mutation was found in the propeller part of the gene among the three cases that were treatment failure (LCF and LPF). However, earlier studies carried out in India did not find C580Y SNP in the *P. falciparum k13* propeller gene [21, 27].

## Conclusion

The high cure rate and parasite clearance time in this study indicate no imminent threat to artemisinin resistance in the regions investigated. As artemisinin derivatives rapidly reduce the symptoms with an additional advantage of anti gametocidal activity to reduce malaria transmission [28]. However, regular monitoring of anti-malaria efficacy is required to satisfy the objectives of the malaria elimination initiative programmes, which are largely based on therapeutic interventions, regular surveillance and targeted vector control. Finally, it is important to conduct detailed phenotyping and genotyping studies for determining artemisinin resistance in such high malarious regions of India.

## Additional file

10.1186/s12936-016-1555-4 Flowchart showing site-wise enrolment and follow-up profile.

## Abbreviations

ACPR: adequate clinical and parasitological response
ACT: artemisinin-based combination therapy
AL: artemether–lumefantrine
AS: artesunate
CHC: Community Health Centre
ETF: early treatment failure
ICMR: Indian Council of Medical Research
*IEC*: Institutional Ethical Committee
LCF: late clinical failure
LFU: lost to follow up
LPF: late parasitological failure
NCBI: National Center for Biotechnology Information
NIMR: National Institute of Malaria Research
NIRTH: National Institute for Research in Tribal Health
NS: non-synonymous mutations
PCR: polymerase chain reaction
SNP: single nucleotide polymorphism
SP: sulfadoxine/pyrimethamine
WBC: white blood cell
WHO: World Health Organization
