# Supplementary figures and images for: Therapeutic efficacy of artemether–lumefantrine for the treatment of uncomplicated Plasmodium falciparum malaria from three highly malarious states in India
Source: Malar J. 2016 Oct 13;15:498. doi: 10.1186/s12936-016-1555-4 (PMC5064902; doi:10.1186/s12936-016-1555-4)

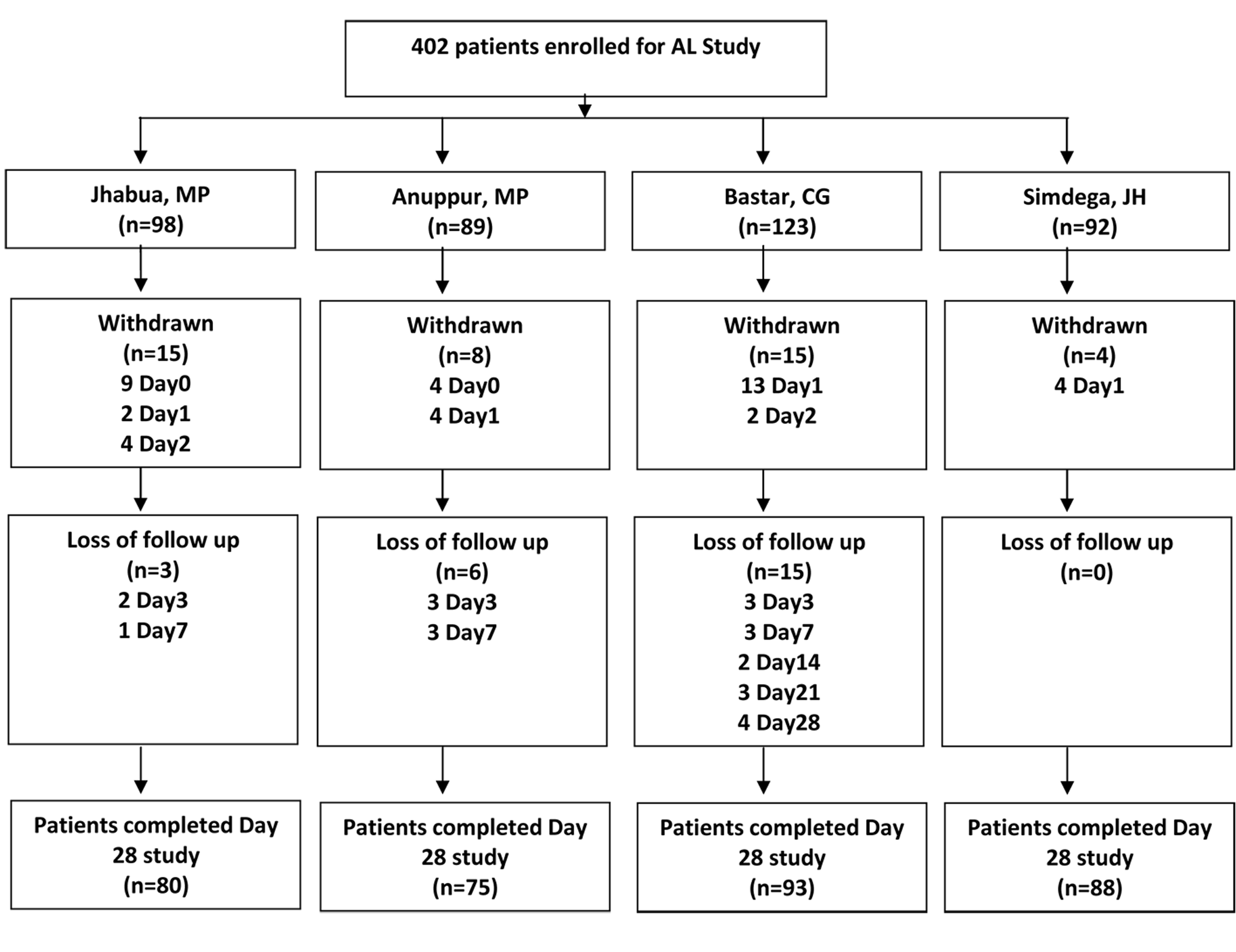

Supplement: Supplementary file 1 — 10.1186/s12936-016-1555-4 Flowchart showing site-wise enrolment and follow-up profile. [file 12936_2016_1555_MOESM1_ESM.tif]
